# Effects of interactive video-game–based exercise on balance in older adults with mild-to-moderate Parkinson’s disease

**DOI:** 10.1186/s12984-020-00725-y

**Published:** 2020-07-13

**Authors:** Rey-Yue Yuan, Shih-Ching Chen, Chih-Wei Peng, Yen-Nung Lin, Yu-Tai Chang, Chien-Hung Lai

**Affiliations:** 1 Department of Neurology, School of Medicine, College of Medicine, Taipei Medical University, Taipei, Taiwan; 2 Department of Neurology, Taipei Medical University Hospital, Taipei, Taiwan; 3 Department of Physical Medicine and Rehabilitation, School of Medicine, College of Medicine, Taipei Medical University, Taipei, Taiwan; 4 Department of Physical Medicine and Rehabilitation, Taipei Medical University Hospital, No. 252, Wu-Hsing St., Taipei City, 110 Taiwan; 5 Taipei Neuroscience Institute, Taipei Medical University, Taipei, Taiwan; 6 School of Biomedical Engineering, College of Biomedical Engineering, Taipei Medical University, Taipei, Taiwan; 7 Graduate Institute of Biomedical Optomechatronics, College of Biomedical Engineering, Taipei Medical University, Taipei, Taiwan; 8 Graduate Institute of Injury Prevention and Control, Taipei Medical University, Taipei, Taiwan; 9 Department of Physical Medicine and Rehabilitation, Wan Fang Hospital, Taipei Medical University, Taipei, Taiwan

**Keywords:** Interactive video game-based exercise, Parkinson’s disease, Balance, Crossover trial

## Abstract

**Background:**

This study aimed to evaluate the effectiveness of a customized interactive video game-based (IVGB) training on balance in older adults with mild-to-moderate Parkinson’s disease (PD).

**Methods:**

In this 12-week crossover trial, PD patients ≥65 years of age were randomly divided into Group A (a 6-week intervention phase followed by a 6-week control phase) and Group B (a 6-week control phase followed by a 6-week intervention phase). Participants received IVGB exercise training during the intervention phase and no exercise during the control phase. Functional outcomes were measured using behavioral evaluation scales and questionnaires at baseline, week 6 and week 12.

**Results:**

Twenty-four PD patients were included in this study, and were evenly divided into two groups. After Bonferroni adjustment, the changes in Modified Falls Efficacy Scale (MFES) and two subscales of Multi-Directional Reach Test were significantly different between two groups in the first 6-week period. In addition, the changes in Berg Balance Scale, MFES, and two subscales of Maximum Step Length were significantly different between two groups in the second 6-week period. Compared to controls, 6-week IVGB exercise intervention significantly improved different but overlapping functional outcomes in two groups of PD patients.

**Conclusions:**

The customized IVGB exercise training improves balance, postural stability and confidence in preventing falls in older adults with mild-to-moderate PD. However, this IVGB exercise doesn’t have a significant impact on quality of life.

**Trial registration:**

ClinicalTrials.gov. NCT03689764. Registered 27 September 2018, retrospectively registered.

## Background

Parkinson’s disease (PD) is the second most common neurodegenerative disease affecting older adults [1]. Neurological deficits in PD affect the musculoskeletal and balance systems, thereby impairing mobility, postural stability, and walking capacity [2, 3]. The clinical symptoms and psychosocial effects of PD often limit the participation of PD patients in social and physical activities, which subsequently further declines in functional status [4]. Restricted mobility in PD patients contributes to secondary health complications and higher treatment costs [5]. Exercise programs benefit PD patients, improving physical strength and function, health-related quality of life, balance, and gait speed to some degree [6, 7]. A cohort study with one-year follow-up revealed that higher frequency of physical activity is associated with a reduced risk of cognitive and motor decline in old adults with mild parkinsonian signs [8]. However, most older people seldom participate in exercise programs because the content of conventional programs may be repetitive, uninviting, or difficult [9]. Hence, it is clinically important to develop an exercise program that is easy and interesting to older adults with mild-to-moderate PD, thereby encouraging patient participation and subsequently ameliorating PD-related motor symptoms.

Auditory and visual biofeedback effectively improves postural stability and balance in healthy adults [10]. Visual cues, combined with treadmill training, result in greater improvements in gait in patients with Hoehn and Yahr (HY) stage 2–4 PD than treadmill training alone [11]. A study of older patients with HY stage 2–3 PD suggested that either auditory or visual cues increase exercise intensity in a virtual cycling system [12]; however, the superiority of auditory over visual cues in ameliorating PD motor symptoms was indicated by a meta-analysis [13]. In addition, a systematic review concluded that gamifying visual feedback and providing performance feedback in real-time facilitate movement rehabilitation in PD [14].

The safety and effectiveness of several commercial exercise gaming (exergaming) systems for motor rehabilitation in PD patients have been extensively investigated [15–21]. Two prospective studies reported that exercises using the virtual-reality–based Wii Fit substantially improved obstacle-crossing performance, dynamic balance, and attention in patients with mild-to-moderate PD (HY stage 3 or lower) [19, 21]. Similarly, two randomized controlled trials demonstrated that PD patients with HY stage 3 or less benefit from commercial exergames during physical rehabilitation [17, 20]. A recent short review found that most of the commercially available rhythm-based games were insufficient for training rhythmic skills, although some features of the games were interesting. Hence, this review concluded that current rhythm-based games still required further modification to devise efficient rhythmic training programs for patients with motor or cognitive impairment [15]. A systematic review published in 2014 concluded that commercial exergames (Nintendo Wii Fit platform and Sony Playstation Eye) often require fast and complex responses that may be too difficult for some PD patients; therefore, specifically tailored exergames are needed to meet the safety and rehabilitation needs of patients with mild-to-moderate PD [16]. A systematic review of PD rehabilitation using commercial exergames in 2014–2017 concluded that exergame-based PD rehabilitation is equal to or more effective than traditional PD rehabilitation [18]. Nevertheless, additional randomized controlled trials (RCTs) that assess more standard outcomes, such as Universal Parkinson’s Disease Rating Scale (UPDRS), Berg Balance Scale (BBS), and Timed Up and Go (TUG) tests, are warranted to confirm the effectiveness of exergames in PD rehabilitation [18].

To meet the unique needs of specific user groups, several customized interactive video-game–based (IVGB) exercise systems have been developed [22–24]. Compared to repetitive conventional physical activities, IVGB training provides an interesting and interactive environment, so participants would be more likely to enjoy completing their physical activity regimen. IVGB exercise is reported to improve dynamic balance control and attention span in patients with traumatic brain injury [22]. Our previous crossover study found that IVGB exercise enabled healthy adults aged ≥65 years to significantly improve scores on the Berg Balance Scale, Modified Falls Efficacy Scale (MFES), and Unipedal Stance Test (UST) and shorten TUG test completion time [24]. In another crossover study, we found that IVGB intervention enhanced BBS scores and reduces TUG task completion time significantly in diabetic patients with peripheral neuropathy [23]. Herein, we hypothesized that IVGB training may improve balance in older adults with mild-to-moderate PD. To examine this hypothesis, the present randomized crossover 12-week trial was conducted in community-living ambulatory elderly patients with mild-to-moderate PD.

## Methods

### Participants

The study cohort included PD outpatients who were recruited from the Neurology Department of Taipei Medical University Hospital, Taiwan. The inclusion criteria were: i) aged 60 to 80 years; ii) clinical diagnosis of idiopathic mild-to-moderate PD of HY stage 1–3 [25]; iii) independent community-living ambulatory individuals; and iv) cognitive level as assessed by the Mini-Mental Status Examination (MMSE) score > 23. The exclusion criteria were: i) history of dementia, previous stroke, arthritis, vision impairment, diabetes, or uremia; ii) previous engagement in any exergaming training program or commercial exergaming system within 6 months; and iii) inability to walk without assistance or the presence of cardiovascular disease that impaired walking.

### Ethical considerations

The study protocol was reviewed and approved by the Joint Institutional Review Board of Taipei Medical University (approval number: TMU-JIRB 201311032). The potential risks and benefits were explained to each participant in advance, and all participants provided signed informed consent before taking part in the current study. The study is registered at ClinicalTrials.gov (identifier number: NCT03689764).

### Study design

**Fig. 1:**
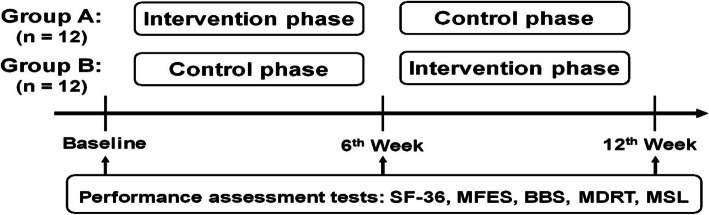
Experimental design. Twenty-four participants with mild-to-moderate PD were randomly assigned to Groups A and B. Participant performance was assessed before intervention (baseline) and at weeks 6 and 12. Group A participated in the interactive video-game-based (IVGB) exercise program during the first 6 weeks, followed by a 6-week period without exercise. Group B participants did the reverse, starting with 6 weeks without exercise followed by 6 weeks of the IVGB exercise program. SF-36, 36-Item Short-Form Health Survey; MFES, Modified Falls Efficacy Scale; BBS, Berg Balance Scale; MDRT, Multi-Directional Reach Test; MSL, Maximum Step Length

In the present prospective, randomized, single-blind, crossover 12-week trial, community-living ambulatory older adults with mild-to-moderate PD were equally divided and randomly assigned to either Group A or Group B. The participants in Group A received IVGB training during the first 6 weeks (intervention phase), followed by no IVGB exercise during the subsequent 6 weeks (control phase); the participants in Group B had no IVGB exercise intervention during the first 6 weeks (control phase) and then received IVGB training in the following 6 weeks (intervention phase) (Fig. 1). Outcomes measured at the end of 6 and 12 weeks were compared between the groups. The crossover design decreased the variability between groups, because the same patients served as the controls and experimental subjects during different time periods as previously described [26].

### IVGB intervention

**Fig. 2:**
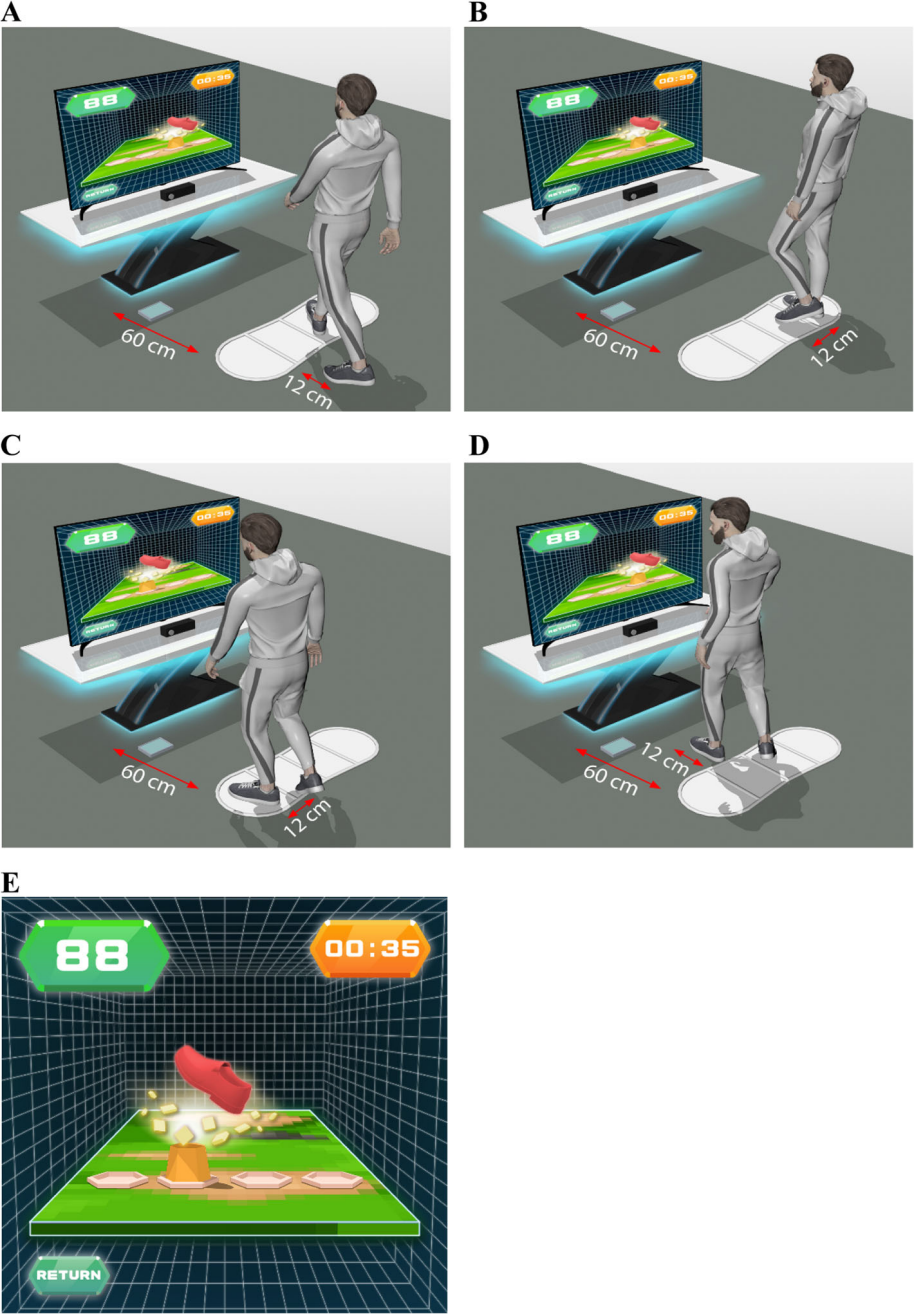
Schematic diagram of the multidirectional reaching task. The stepping mat was placed 60 cm in front of the monitor. **a**: Participants performed interactive multidirectional reaching tasks by following the target appearing on the television screen, with movements tracked by infrared photosensors in the controllers. **b** & **c**: For the antero-posterior stepping practice, participants stood 12 cm away from the mat in front/behind and stepped onto the target area according to the monitor cues. **d** & **e**: For the medial-lateral stepping practice, participants stood on the right/left side of mat (starting area) and stepped onto the target area according to the monitor cues. For forward–backward and medial–lateral stepping, the floor and mat were marked to ensure that participants started and ended in the same place

**Fig. 3:**
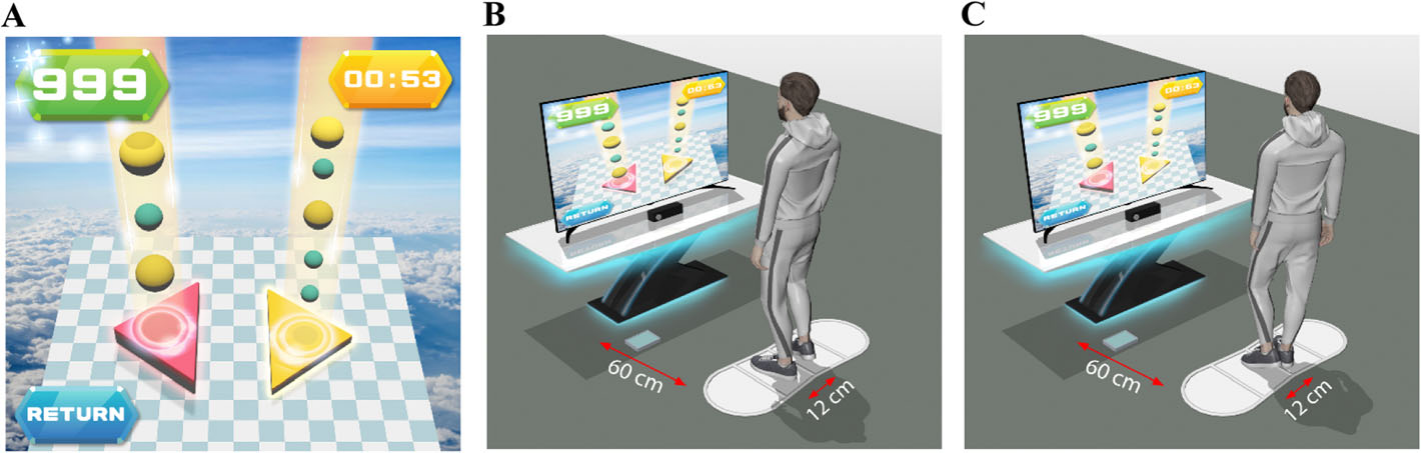
Schematic diagram of the target-oriented stepping task. The stepping mat was placed 60 cm in front of the monitor. Participants stood on the marked area of the mat as the starting point. **a**: Participants performed the interactive target-oriented stepping task according to the cue on the television screen, with motion monitored by infrared photosensors in the controllers. **b**: Right foot was raised according to the cue on the monitor. **c**: Left foot was raised according to the cue on the monitor

The IVGB system was developed by modifying the XaviX entertainment system (SSD Company Limited, Shiga, Japan). We previously reported the beneficial effects of the IVGB system on motor recovery [23, 24]. The XaviX system was originally designed for healthy users. Hence, we modified the difficulty levels and scoring system of the XaviX system for older adults with mild-to-moderate PD, and the step mat was fixed on the floor to minimize the risk of falls. The IVGB exercise program consisted of two tasks: a multi-directional step task and a target-directed stepping task. The IVGB system offers aural and visual feedback in both tasks to increase participants’ attention. First, the participant followed the illustrated instructions shown on the monitor to step on the target area to complete the multi-directional step task (Fig. 2). This first task assesses the participant’s capability for weight-shifting, dynamic balance, and stability. The participant then followed the illustrated instruction to complete the target-directed stepping task (Fig. 3). This second task evaluates the participant’s movement coordination and balance while standing on one leg. Adjustments were made between three levels of difficulty and direction of steps by a physical therapist in accordance with the participant’s cognition, attention, balance, walking ability, strength, and weight-shifting ability. To ensure uniformity in exercise posture, participants were asked to maintain an upright position and to avoid compensating by postural sway. All IVGB training sessions were held at Taipei Medical University Hospital and were instructed and monitored by a certified physical therapist who was responsible for ensuring participant safety during the exercises.

Exercise performance measurements, including time to complete, number of successful steps, and avatar-mimicking foot trajectory, were displayed on the monitor in real time during IVGB training and summarized at the end of the training. During the 6-week training (intervention phase), participants reported to the hospital 3 days each week to complete the 30-min training (15 min per task).

### Outcome measures

Outcome measures were assessed at the end of the first 6-week intervention/control phase and the end of the subsequent 6-week intervention/control phase to determine the effects of IVGB training (Fig. 1). The primary outcome measure was the BBS score; the secondary outcome measures included scores on the 36-Item Short-Form Health Survey (SF-36), MFES, Multi-Directional Reach Test (MDRT), and Maximum Step Length (MSL) test.

#### BBS

The BBS objectively assesses balance ability via 14 functional balance tasks, and has been confirmed effective in PD patients [27]. The BBS applies a 4-point scale, in which a score of 0 indicates inability to perform the task and a score of 4 indicates task completion. A total BBS score less than 46 points indicates the risk of falls [28].

#### SF-36

The SF-36 is a self-reported quality of life questionnaire that assesses health status of 8 domains, including physical functioning (10 items), social functioning (2 items), physical problems (4 items), emotional problems (3 items), general mental health (5 items), vitality (4 items), bodily pain (2 items), and general health (5 items). Each item is scored from 0 (worst Health) to 100 (best health) [29]. The SF-36 has been wildly used to evaluate the impact of PD on quality of life [30, 31].

#### MFES

The MFES is a 14-item questionnaire regarding daily indoor and outdoor physical activities and is a valid tool for measuring fall-related self-efficacy in PD [32].

#### MDRT

The MDRT is a valid assessment of stability in multiple directions [33]. The participants were required to reach in the forward, left, and right directions. For the forward-reaching direction, the participant raised both arms to the shoulder level, reached forward as far as possible without moving the feet, and then maintained the end-range position for 3 s. The distance reached by the middle finger was measured. Similar methods were used to assess right and left lateral reach. The assessment was performed 3 times in each direction, and the data were averaged for further statistical analysis.

#### MSL

The MSL test is a valid measurement of stepping ability and serves as an indicator of mobility function and fall risk in older adults [34]. Participants performed the MSL test in the forward, lateral, and backward directions. For the forward direction test, the participant crossed the arms over the chest, took a maximum step forward with one leg only, and then returned to the starting position with a single step. The stepping distance was measured. The same method was used the lateral and backward direction tests.

### Statistical analysis

Continuous variables are presented as the mean ± standard deviation (SD), and the corresponding between-group differences were analyzed using Student’s t-test. Categorical variables are presented as the count and percentage, and the corresponding between-group differences were analyzed using the chi-square test or Fisher’s exact test. Two-tailed *P* < 0.05 indicated statistical significance for the baseline characteristic analysis. To test for differences in outcome measures between time points (baseline, week 6 and week 12), repeated-measures ANOVA was performed separately for two different patient groups. To examine difference in changes in outcome measures between Group A and Group B, t-test was performed separately for two distinct 6-week periods. Since SF-36, MDRT and MSL contain multiple subscales, *P* values of the subscales of SF-36, MDRT and MSL were corrected using the Bonferroni method. The alpha value was set to *P* = 0.05/ (number of subscales x number of categories); categories could be time points or patient groups in the present study. Hence, the Bonferroni correction was conducted in between-time point comparison of each subscale in the same group, and in between-group comparison of the change in each subscale in the same 6-week period. All statistical analyses were performed using SAS version 9.4 (Windows NT version, SAS Institute, Inc., Cary, NC, USA).

## Results

A total of 24 community-living older adults with mild-to-moderate PD were included in this prospective crossover study and were randomly divided into two groups: Group A and Group B (Fig. 1). The demographic and clinical characteristics of two groups of PD patients are shown in Table 1. Age, body height, body weight, body mass index (BMI), leg length, foot length, fall in the past year, and HY stage were comparable between two groups. However, the MMSE score and percentage of females were significantly lower in Group B than in Group A (Table 1).

**Table 1:** Baseline demographic and clinical characteristic of two groups of PD patients

	Group A	Group B	*P*-value
	*N* = 12	N = 12	
	Mean ± SD		
Age (years)	67.8 ± 5.5	66.5 ± 8.8	0.66^a^
Body height (cm)	158.5 ± 7.4	164.3 ± 7.5	0.07^a^
Body weight (kg)	58.0 ± 10.9	61.1 ± 6.3	0.42^a^
BMI (kg/m^2^)	23.2 ± 4.6	22.7 ± 2.4	0.75^a^
Leg length (cm)			
Left leg	79.5 ± 4.5	81.1 ± 5.2	0.44^a^
Right leg	79.6 ± 4.8	81.2 ± 5.6	0.46^a^
Foot length (cm)			
Left foot	23.3 ± 1.1	24.2 ± 1.6	0.10^a^
Right foot	23.3 ± 1.1	24.2 ± 1.6	0.11^a^
MMSE	28.5 ± 1.7	26.0 ± 2.6	**0.01**^a^
	N (%)		
Sex			
Female	10 (83.33%)	3 (25%)	**0.004**^b^
Male	2 (16.67%)	9 (75%)	
Fall in the past year			
No	12 (100%)	8 (66.67%)	0.09^c^
Yes	0 (0%)	4 (33.33%)	
Hoehn and Yahr staging scale			
Stage 1	4 (33.33%)	3 (25%)	0.77^c^
Stage 2	5 (41.67%)	4 (33.33%)	
Stage 3	3 (25%)	5 (41.67%)	

*SD* standard deviation, *N* number, *%* percentages, *BMI* Body mass index, *MMSE* Mini-mental state examination

^a^T Test; ^b^ Chi-square Test; ^c^ Fisher’s Exact Test

Significant difference (*p* < 0.05) was indicated in bold

**Fig. 4:**
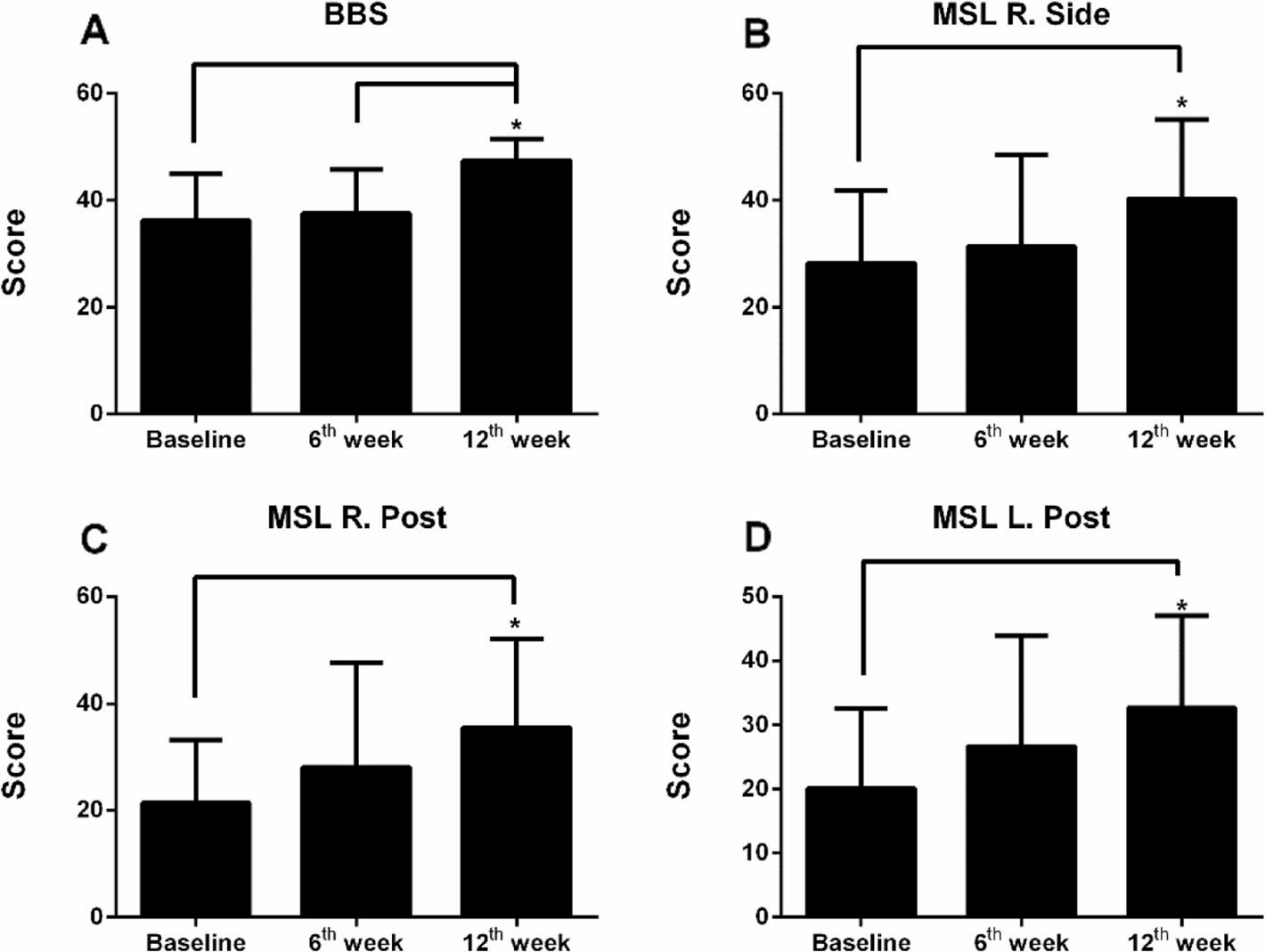
Outcome measures collected at three time points in Group B. The scores were presented as mean ± SD. Differences between time points were examined using repeated measures ANOVA, followed by a post hoc *Bonferroni* test. The alpha value was set to 0.05 for BBS and MFES; 0.0021 for subscales of SF-36; 0.0056 for subscales of MDRT; 0.0028 for subscales of MSL

The outcome measures of the two groups at various time points are shown in Supplemental Table 1. After the Bonferroni correction, no significant differences in all outcome measures between time points were found in Group A. In contrast, in Group B, BBS at week 12 was significantly higher than those at baseline and at week 6 (Fig. 4a). In addition, three subscales of MSL, including the right side (R. Side), the posterior side of the right leg (R. Post) and the posterior side of the left leg (L. Post), were significantly higher at week 12 than at baseline (Fig. 4b-d).

In addition, between-group comparison of the changes in outcome measures in the first 6-week period (values at week 6 minus values at baseline) and in the second 6-week period (values at week 12 minus values at week 6) were conducted separately (Table 2). After the Bonferroni correction, the changes in MFES and two subscales of MDRT, including MDRT to the right side (MDRT-R) and MDRT-L, were significantly different between two groups in the first 6-week period. Moreover, the changes in BBS, MFES, and two subscales of MSL (R. Post and L. Post) were significantly different between two groups in the second 6-week period (Table 2).

**Table 2:** Between-group comparisons of the changes in outcome measures in the first and second 6-week periods

	First 6-week period			Second 6-week period		
	Group A	Group B	*P* value ^a^	Group A	Group B	*P* value ^a^
	Mean ± SD			Mean ± SD		
BBS (score)	1.0 ± 3.8	1.4 ± 6.7	0.85	−3.1 ± 4.9	9.9 ± 7.2	**< 0.0001**
SF-36 (score)						
Physical functioning	9.6 ± 10.5	0 ± 19.2	0.14	2.5 ± 13.6	10.9 ± 19.1	0.23
Physical problems	10.0 ± 31.3	−13.9 ± 32.4	0.09	−1.7 ± 23.9	18.8 ± 28.5	0.07
Emotional problems	10.9 ± 29.5	−23.3 ± 39.5	0.02	−2.9 ± 25.2	29.6 ± 35.2	0.02
Vitality (energy/ fatigue)	13.8 ± 13.5	2.9 ± 25.1	0.20	−0.4 ± 21.4	8.3 ± 23.1	0.35
General mental health	11.4 ± 21.5	5.3 ± 14.3	0.42	−0.2 ± 25.0	5.8 ± 22.5	0.54
Social functioning	14.2 ± 34.2	0.6 ± 19.6	0.25	0 ± 0	11.3 ± 28.2	0.19
Bodily pain	5.6 ± 31.6	15.2 ± 26.9	0.43	1.7 ± 35.5	9.6 ± 29.2	0.56
General health	6.7 ± 34.7	7.9 ± 18.1	0.91	4.6 ± 35.3	8.8 ± 20.6	0.73
MFES (score)	8.3 ± 17.0	−8.1 ± 14.1	**0.02**	−6.5 ± 15.0	21.3 ± 35.7	**0.03**
MDRT (cm)						
MDRT-F	1.6 ± 6.5	3.1 ± 6.5	0.57	−1.7 ± 6.2	1.2 ± 6.5	0.29
MDRT-R	8.5 ± 7.4	−0.5 ± 5.5	**0.003**	−2.7 ± 8.6	2.2 ± 4.2	0.09
MDRT-L	6.9 ± 5.9	−0.8 ± 5.8	**0.004**	−2.7 ± 8.3	3.6 ± 6.2	0.05
MSL (cm)						
R. Ant	3.1 ± 10.4	4.9 ± 12.4	0.69	−2.3 ± 8.2	2.7 ± 6.8	0.12
L. Ant	2.4 ± 12.4	2.8 ± 4.4	0.92	−1.2 ± 9.7	4.6 ± 8.3	0.13
R. Side	2.6 ± 8.7	3.1 ± 9.4	0.89	−1.8 ± 9.7	8.9 ± 10.4	0.02
L. Side	0.5 ± 10.2	−0.03 ± 7.3	0.89	−1.4 ± 11.0	7.5 ± 9.1	0.04
R. Post	4.3 ± 13.4	6.7 ± 8.9	0.63	−6.4 ± 9.3	7.5 ± 11.5	**0.004**
L. Post	2.9 ± 14.3	6.7 ± 7.7	0.43	−5.9 ± 8.3	5.9 ± 9.2	**0.003**

*SD* standard deviation, *BBS* Berg Balance Scale, *SF-36* the 36-Item Short-Form Health Survey, *MFES* Modified Falls Efficacy Scale, *MDRT* Multi-Directional Reach Test, *MDRT-F* MDRT to the forward side, *MDRT-R* MDRT to the right side, *MDRT-L* MDRT to the left side, *MSL* Maximum Step Length, *R. Ant* right leg, anterior side, *L. Ant* left leg, anterior side, *R. Side* right side, *L. Side* left side, *R. Post* right leg, posterior side, *L. Post* left leg, posterior side

^a^The alpha value was set to 0.05 for BBS and MFES; 0.0031 for subscales of SF-36; 0.0083 for subscales of MDRT; 0.0042 for subscales of MSL. Significant difference after Bonferroni correction was indicated in bold

In contrast to BBS, MFES, MDRT and MSL, IVGB training did not significantly altered any subscale of SF-36 (Table 2). In addition, no adverse effects were observed during the entire study period.

## Discussion

In this prospective crossover study, we found that a 6-week IVGB exercise program significantly enhanced confidence in preventing falls, as evidenced by higher scores on MFES, in all included older outpatients with mild-to-moderate PD. In addition, 6-week IVGB training significantly improved the ability of balance and the capability of stepping their leg in the left, right and back directions in patients undergoing IVGB training in the second period. However, no significant impact of IVGB training on quality of life was found in this study. Thus, IVGB intervention significantly improved different but overlapping functional outcomes in two groups of older patients with mild-to-moderate PD.

In the present study, IVGB training significantly improved BBS in the second 6-week period, but not in the first 6-week period, which might be in part due to the fact that the mean baseline BBS score of the PD patients undergoing IVGB training in the second period was lower than that of those undergoing IVGB training in the first period (36.2 ± 8.9 vs. 50.7 ± 3.5). Supporting our speculation, a recent study reported that PD patients with lower baseline BBS scores achieve greater improvements in balance rehabilitation that those with higher BBS scores do [35]. In addition, improvements in balance using conventional balance training are suggested to be influenced by cognitive reserve, an individual-specific active expression of the brain’s ability to respond to physical damage [35]. In the present study, compared to PD patients undergoing IVGB training first, PD patients undergoing IVGB training in the second period had more male patients but a lower mean MMSE score, which might also partially contribute to the different results between groups. Hence, additional studies with a larger sample size are warranted to investigate the extent to which sex and mental status affect the effectiveness of IVGB training.

Depression and anxiety are commonly noted in PD patients, and both are highly associated with worsening motor function, symptom fluctuations, cognitive losses, and poor quality of life [36]. Game-based exercise training provides not only physically challenging exercise but also cognitive stimulation, thereby boosting both physical and mental performance [37]. A systematic review concluded that exergaming provided an innovative, fun, and relatively safe method of exercise and had significant effects on physical and cognitive function in persons with dementia [38]. The present study demonstrated that 6-week IVGB training significantly elevated MFES scores in all included PD patients, indicating that PD patients’ confidence in avoiding falls was boosted after IVGB training. Such beneficial effect of IVGB training on confidence in avoiding falls has been previously suggested in healthy adults aged ≥65 years [24] and in diabetic patients with peripheral neuropathy [23]. However, IVGB training did not significantly alter all subscales of SF-36 in the present study, indicating no obvious impact of IVGB training on quality of life in older patients with mild-to-moderate PD.

The stepping reaction, which is critical for successful recovery of balance, requires the integration of neural and sensorimotor control systems [39]. Previous studies indicated that stepping exercises on a treadmill and tango dancing increased balance and mobility in PD patients [39, 40]. In addition, PD patients perform lateral weight shifting with less accuracy than do healthy controls [41]. The intervention using weight-shifting balance exercises significantly increases postural stability in the anterior-posterior and medial-lateral directions, which is commonly limited in PD patients [42]. It has been demonstrated that randomized rightward, leftward, and forward-backward perturbations improved protective postural responses in PD patients [43]. Consistently, we observed in the present study that multi-directional step task and target-directed stepping task of the IVGB exercise program improved motor coordination, the ability to stand on one leg, and functional stepping in older patients with mild-to-moderate PD, although not all subscales of MDRT and MSL were improved by IVGB training in all included PD patients.

The current customized IVGB exercise program may provide several benefits to older patients with mild-to-moderate PD. First, the included PD patients indicated that IVGB training with auditory and visual feedback more interesting, compared to conventional stepping and balance training. In addition, the real-time feedback regarding exercise performance during IVGB training gives PD patients a sense of achievement, thereby motivating them to do their best. Finally, the IVGB training program consists of the multiple direction stepping task and target-oriented stepping tasks of the IVGB training program address balance, postural control, and weight-shifting ability; therefore, both tasks are suitable for motor rehabilitation in PD.

The benefits of virtual-reality–based physical exercises (exergames) in PD motor rehabilitation have been long demonstrated. Treadmill training with virtual obstacles improves physical performance and gait in older persons with PD [44]. Physical rehabilitation using a customized videogame ameliorates gait and balance problems in PD patients [45]. Furthermore, the Wii Fit Exercise substantially enhances obstacle-crossing performance and dynamic balance in patients with HY stage 1–3 PD [19], and similar promising results were reported in two RCTs [17, 20]. On the other hand, the superiority of auditory cueing over visual cueing in PD rehabilitation has been suggested [13], and rhythmic auditory stimulation reinforced the beneficial effect of multidirectional step training on gait and balance in PD [46]. Rhythmic skills are suggested to serve as a predictor of disease severity and recovery capacity upon auditory cueing in patients with HY stage 2 PD [47], and rhythm-based games (music games) enhance rhythmic skills and motor functions in PD patients [48]. Therefore, it is reasonable to assume that the integration of rhythmic stimulation (music) into the current IVGB exercise program may further strengthen its effectiveness in motor recovery in older patients with mild-to-moderate PD.

A recent study reported that a home-based, square-stepping exercise increased mobility and cognitive skills among older adults with multiple sclerosis [9]. Furthermore, a 6-week training using a music-based game at home improves rhythmic skills in patients with HY stage 2–3 PD [48]. In the present study, all IVGB training courses were held at a hospital in the presence of a physical therapist who was responsible for preventing falls. Further prospective studies with a larger sample size are warranted to evaluate the feasibility of home-based IVGB training for PD patients; to determine the appropriate users in terms of age, disease severity, and comorbidities; and to develop relevant safety guidelines.

Although the physiological and neural mechanisms underlying the functional benefits of exergames remain to be investigated, the notion that exercise boosts synaptic strength and potentiates functional circuity in PD is widely accepted [49]. In addition, several possible neurobiological mechanisms have been proposed to underlie this relationship, including increased release of neurotransmitters, modulators, and tropic factors, as well as cognitive gains [50].

The present study had several limitations. First, the sample size was small. Second, the sex ratio and the mean MMSE score were unequal between the two groups, which might confound the results to some degree. Third, physiological variables such as heart rate and pulse rate were not assessed in this study. Finally, conventional motor rehabilitation was not included for comparison. Further investigation with a larger population of age-, sex-, mental status- and HY-stage-matched PD patients and longer follow-up periods is needed to confirm the effectiveness of IVGB training over that of conventional physiotherapy in PD rehabilitation.

## Conclusion

A 6-week, hospital-based IVGB exercise program improved the balance, postural stability, and confidence in preventing falls in older patients with HY stage 1–3 PD. Thus, IVGB exercise training might serve as a rehabilitation regimen to ameliorate physical symptoms in older adults with mild-to-moderate PD.

## Supplementary information

**Additional file 1: Supplemental Table 1**. Outcome measures of two groups of PD patients at baseline, week 6 and week 12.

## Data Availability

The datasets generated during the current study are available from the corresponding author upon request.

## Abbreviations

PD: Parkinson’s disease
IVGB: Interactive video game-based
BBS: Berg Balance Scale
MFES: Modified Falls Efficacy Scale
UST: Unipedal Stance Test
TUG: Timed Up and Go
MMSE: Mini-Mental Status Examination
SF-36: 36-Item Short-Form Health Survey
MDRT: Multi-Directional Reach Test
MSL: Maximum Step Length
Mean ± SD: Mean and standard deviation
BMI: Body Mass Index
MDRT-F: Multi-Directional Reach Test to the front side
MDRT-R: Multi-Directional Reach Test to the right side
MDRT-L: Multi-Directional Reach Test to the left side
RCTs: Randomized controlled trials
