# Supplementary material for: Effects of interactive video-game–based exercise on balance in older adults with mild-to-moderate Parkinson’s disease
Source: J Neuroeng Rehabil. 2020 Jul 13;17:91. doi: 10.1186/s12984-020-00725-y (PMC7359629; doi:10.1186/s12984-020-00725-y)
Supplement: Supplementary file 1 — Additional file 1: Supplemental Table 1. Outcome measures of two groups of PD patients at baseline, week 6 and week 12. [file 12984_2020_725_MOESM1_ESM.docx]

Supplemental Table 1. Outcome measures of two groups of PD patients at baseline, week 6 and week 12.

|  |  | Group A | | |  | Group B | | | |
| --- | --- | --- | --- | --- | --- | --- | --- | --- | --- |
|  |  | Baseline | 6^th^ week | 12^th^ week |  | | Baseline | 6^th^ week | 12^th^ week |
|  | | Mean ± SD | | |  | | Mean ± SD | | |
| BBS (score) | | 50.7±3.5 | 51.7±2.8 | 48.6±6.3 |  | | 36.2±8.9 | 37.6±8.2 | 47.5±4.1 |
| SF-36 (score) | |  |  |  |  | |  |  |  |
| Physical functioning | | 59.6±24.3 | 69.2±19.5 | 71.7±16.6 |  | | 43.8±30.8 | 43.8±21.2 | 54.7±21.8 |
| Physical problems | | 63.8±29.4 | 73.8±20.0 | 72.1±16.4 |  | | 29.6±43.5 | 16.7±30.8 | 35.4±44.5 |
| Emotional problems | | 63.3±30.2 | 74.3±21.8 | 71.4±19.2 |  | | 47.6±40.9 | 24.3±32.1 | 53.9±44.3 |
| Vitality (energy/fatigue) | | 53.3±22.9 | 67.0±22.7 | 66.7±23.5 |  | | 42.1±27.0 | 45.0±17.8 | 53.3±24.3 |
| General mental health | | 54.8±27.7 | 66.3±26.8 | 66.1±24.1 |  | | 49.4±24.1 | 54.7±18.2 | 60.5±17.6 |
| Social functioning | | 53.8±31.4 | 67.9±31.4 | 67.9±31.4 |  | | 58.3±33.4 | 58.9±24.9 | 70.3±23.6 |
| Bodily pain | | 56.7±35.4 | 62.3±36.1 | 63.9±30.1 |  | | 55.2±30.9 | 70.4±23.6 | 80.0±17.1 |
| General health | | 49.2±32.1 | 55.8±33.9 | 60.4±31.9 |  | | 35.0±21.9 | 42.9±11.6 | 51.7±19.6 |
| MFES (score) | | 115.8±21.7 | 124.1±21.3 | 117.6±31.9 |  | | 79.6±41.6 | 71.5±41.2 | 92.8±24.9 |
| MDRT (cm) | |  |  |  |  | |  |  |  |
| MDRT-F | | 19.2±6.1 | 20.8±5.8 | 19.0±3.7 |  | | 13.8±4.6 | 16.9±7.1 | 18.1±5.4 |
| MDRT-R |  | 14.4±4.9 | 22.9±6.6 | 20.2±7.9 |  | | 14.1±6.7 | 13.6±6.5 | 15.8±5.0 |
| MDRT-L |  | 15.9±4.6 | 22.8±4.9 | 20.1±7.5 |  | | 13.6±7.3 | 12.8±5.9 | 16.4±6.4 |
| MSL (cm) |  |  |  |  |  | |  |  |  |
| R. Ant | | 57.3±13.8 | 60.4±11.9 | 58.1±14.2 |  | | 28.5±18.7 | 33.4±19.4 | 36.1±17.9 |
| L. Ant | | 57.9±13.9 | 60.3±9.8 | 59.1±13.3 |  | | 28.0±17.4 | 30.9±18.8 | 35.5±15.7 |
| R. Side | | 60.4±11.5 | 63.0±13.9 | 61.2±13.2 |  | | 28.2±13.6 | 31.4±17.2 | 40.3±14.9 |
| L. Side | | 61.9±11.7 | 62.4±11.1 | 61.1±11.3 |  | | 31.9±18.4 | 31.9±17.9 | 39.4±17.5 |
| R. Post | | 53.6±17.4 | 57.9±15.7 | 51.5±16.4 |  | | 21.4±11.8 | 28.0±19.6 | 35.5±16.6 |
| L. Post | | 52.6±17.5 | 55.4±14.9 | 49.5±15.7 |  | | 20.1±12.5 | 26.7±17.2 | 32.7±14.3 |

SD: standard deviation; BBS: Berg Balance Scale; SF-36: the 36-Item Short-Form Health Survey; MFES: Modiﬁed Falls Efﬁcacy Scale; MDRT: Multi-Directional Reach Test; MDRT-F: MDRT to the forward side; MDRT-R: MDRT to the right side; MDRT-L: MDRT to the left side; MSL: Maximum Step Length; R. Ant: right leg, anterior side; L. Ant: left leg, anterior side; R. Side: right side; L. Side: left side; R. Post: right leg, posterior side; L. Post: left leg, posterior side.
